# Horses’ rejection behaviour towards the presence of *Senecio jacobaea* L. in hay

**DOI:** 10.1186/s12917-021-03124-0

**Published:** 2022-01-07

**Authors:** Louisa Sroka, Clara Müller, Marie-Lena Hass, Anja These, Sabine Aboling, Ingrid Vervuert

**Affiliations:** 1 Institute of Animal Nutrition, Nutrition Diseases and Dietetics, Faculty of Veterinary Medicine, Leipzig University, Leipzig, Germany; 2 Institute for Animal Nutrition, University of Veterinary Medicine Hannover, Hannover, Germany; 3 Department Safety in the Food Chain, German Federal Institute for Risk Assessment, Berlin, Germany

**Keywords:** Selection behaviour, Pyrrolizidine alkaloids, Toxic plants, Ad libitum, Tansy ragwort

## Abstract

**Background:**

*Senecio jacobaea* contains pyrrolizidine alkaloids that can induce severe hepatic intoxication in horses, either acute when ingested in high amounts or chronic when consumed over a long period. The aim of this study was to determine horses’ rejection behaviour towards the presence of *Senecio jacobaea* in hay when fed ad libitum. We hypothesized that adult horses can sort *Senecio jacobaea* out of the contaminated hay when hay is fed ad libitum. Six warmblood geldings with a mean (±SD) age of 15 ± 2 years were included. In a randomized study, *Senecio jacobaea* contaminated hay (5% or 10% contamination level) was provided at several timepoints over the day for 1 hour to six. Hay was provided ad libitum for the rest of the day. The horses’ rejection behaviour towards *Senecio jacobaea* was observed. If a horse ingested two *Senecio jacobaea* plants twice at different timepoints, then the horse was excluded from the experiment.

**Results:**

Two out of six horses had to be excluded from the study after three out of 12 observation periods due to repeated *Senecio jacobaea* intake. Two other horses had to be excluded after nine and 11 out of 12 observation periods. Only two horses were able to sort out the various amounts (5 and 10% contamination level) of *Senecio jacobaea* during the whole experiment.

**Conclusions:**

Horses’ intake of *Senecio jacobaea* cannot be avoided despite being fed with hay ad libitum. Due to the risk of chronic intoxication by pyrrolizidine alkaloids intake, feeding *Senecio jacobaea* contaminated hay must be avoided, and pastures with *Senecio jacobaea* growth are considered inappropriate for feed production.

## Background

*Senecio jacobaea* L. (SJ, family: *Asteraceae*, genus: *Senecio*, syn. Tansy ragwort) [1, 2] is a toxic plant on humus-enriched, sandy, loam, and clay soils, which can be found worldwide [1, 3, 4]. SJ contains pyrrolizidine alkaloids (PAs), which are secondary plant metabolites [5] that can lead to severe intoxication in horses and other animals [6]. Acute intoxication by SJ intake is possible but rarely reported in horses. However, the accidental intake of small quantities of SJ over a long period can lead to PA accumulation in liver tissue and may induce severe chronical intoxication such as seneciosis in farm animals [4, 6]. The most common clinical symptoms of chronic PA intoxication include icterus, photodermatitis, neurological deficits, and ataxia [6–9]. In recent years, SJ has increasingly spread due to the following factors: 1) increase in fallow land, 2) increased nitrogen deposition in the air, 3) purpose seeding in roadside greenery, and 4) climate changes such as drought [10]. These factors have led to an increasing plant population on pasture and grassland, which may result in SJ-contaminated hay [1, 10, 11].

After references [4–6] it is described that horses usually avoid SJ probably on pasture due to the bitter taste. In contrary to the rejection behaviour of horses on pasture, the ability to reject SJ in hay is rarely reported in horses. However, the intake of SJ-contaminated hay may be associated with a high health risk especially in combination with restricted forage supply due to hay shortages during the last years [12]. To the best of our knowledge no clinical studies to this topic were performed in horses.

Due to this the study aimed to monitor horses’ rejection behaviour (5 and 10% contamination level) in relation to the presence of SJ in hay when provided ad libitum. We hypothesized that adult horses could sort SJ out of the contaminated hay when hay is fed ad libitum.

## Results

### Health monitoring

#### Clinical examinations

Five of the six horses did not show any abnormalities during the regular examinations. At day seven of the experiment, one horse showed colic symptoms. After medical colic treatment, the horse recovered within 2 days.

#### Blood analysis

Selected blood parameters at the beginning and at the end of the study are summarized in Table 1. All blood parameters were within the reference ranges.

**Table 1:** Blood parameters at the beginning and at the end of the experiment and reference ranges

Blood parameters	Start of the feeding period	End of the feeding period	*P*-value	Reference parameters [13]
White blood cells (G/L)	6.4 ± 1.27	6.55 ± 0.836	0.701	4.4-12
Total protein (g/L)	66.2 ± 5.98	68.8 ± 5.74	0.015	57.8-78.7
Albumin (g/L)	33 ± 2.1	35.3 ± 1.34	0.025	27.3-37.0
Cholesterol (mmol/L)	2.29 ± 0.347	2.21 ± 0.267	0.326	1.72-2.95
Bilirubin (μmol/L)	22.6 ± 5.87	24.9 ± 1.7	0.355	15.1-47.0
BA (μmol/L)	3.77 ± 0.845	4.98 ± 1.38	0.094	< 12
TAG (mmol/L)	0.325 ± 0.056	0.288 ± 0.041	0.272	0.13-0.61
AST (U/L)	369 ± 44.35	367 ± 43.55	0.948	213-627
ƴGT (U/L)	27.1 ± 7.09	20.55 ± 7.78	0.038	6.39-44.8
GLDH (U/L)	2.02 ± 0.293	2.92 ± 1.79	0.269	1.39-11.41
LDH (U/L)	381 ± 78.8	349 ± 55.7	0.087	224-536

Data are expressed as mean ± SD. *BA* bile acid, *TAG* triacylglycerol, *AST* aspartate aminotransferase, *ƴGT* gamma-glutamyl transferase, *GLDH* glutamate dehydrogenase, *LDH* lactate dehydrogenase

### Observation periods

**Fig. 1:**
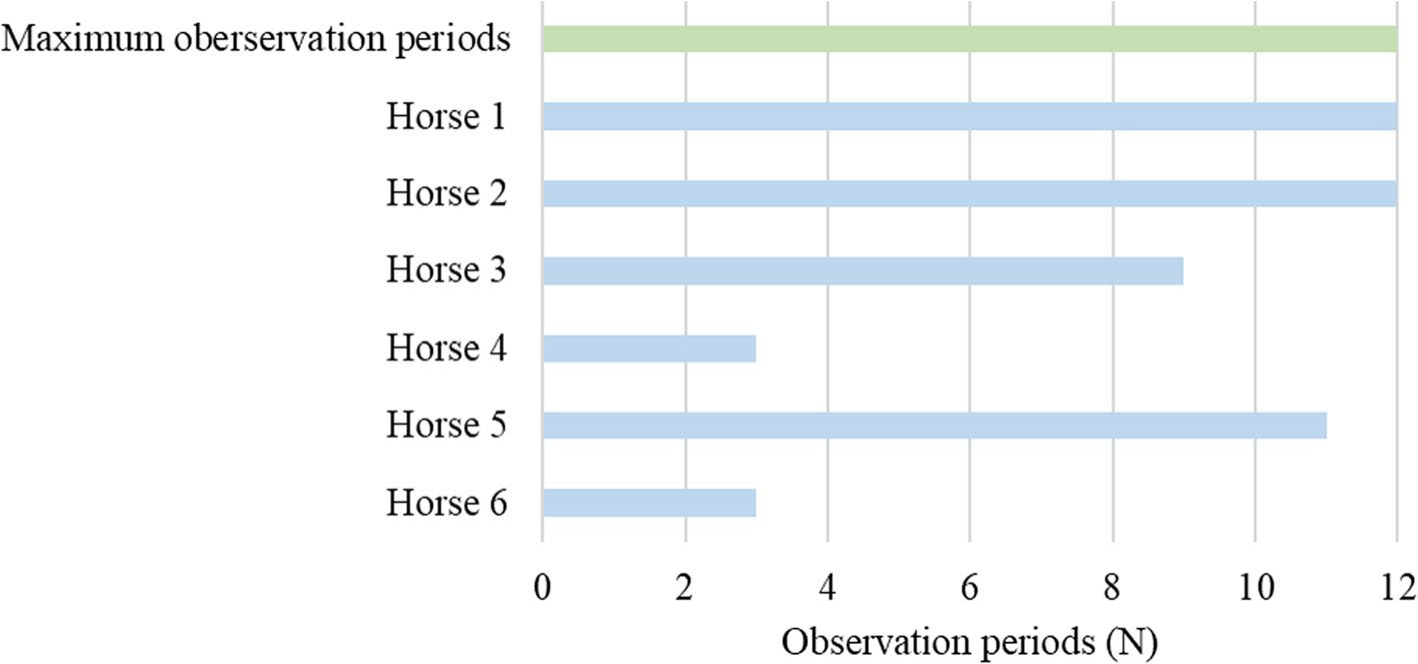
Observation periods of the horses in relation to the maximum possible observation periods (*N* = 12 observation periods per horse). Observation periods below 12 denote an interruption of feeding experiment due to SJ ingestion. Data are expressed individually

Two out of six horses ingested SJ within the first three observation periods and had to be excluded from the experiment. Another two horses showed an inconsistent rejection behaviour. On some days, the horses ingested stems of variable size, while on other days, the horses were able to sort out SJ independently of the size of the stems. These two horses had to be excluded after nine and 11 observation periods. Two horses were able to reject the various amounts of SJ (5 and 10% contamination level) throughout the whole feeding period (Fig. 1).

**Fig. 2:**
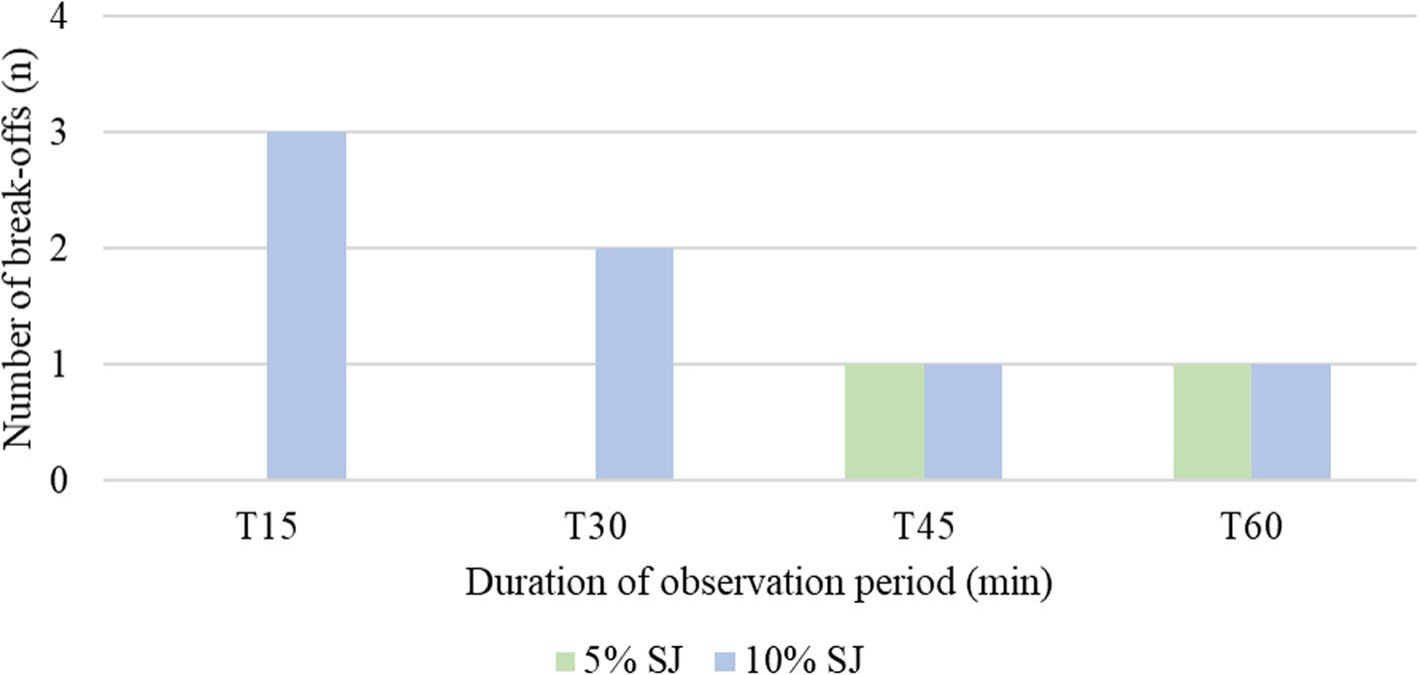
Number of breakoffs in relation to the duration of observation periods (T = time in minutes), breakoffs in total *n* = 9

Break-offs of the observation periods by ingestion of SJ by the respective horse were evenly distributed over the 60 min (Fig. 2).

### Toxin analysis

**Fig. 3:**
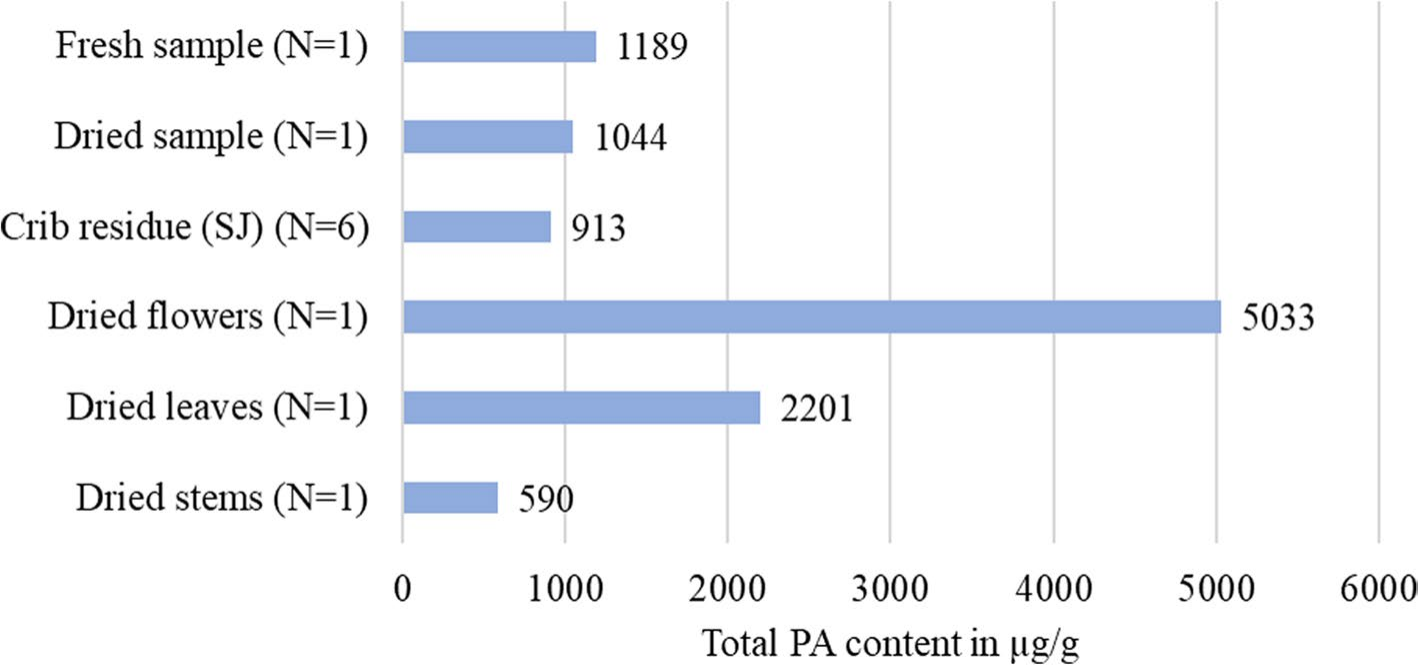
Total PA contents in whole fresh plants or dried plant material. SJ in crib residues and in individual parts such as dried flowers, leaves, and stems. Data are expressed in μg/g (DM)

The total PA content is determined as the sum of individual alkaloids in dry matter (DM). The PA content was highest in dried flowers, followed by leaves. Stems had the lowest content. PA-content in fresh plant material (freeze-dried) was only slightly higher than in dried plant material (Fig. 3).

### Crude nutrients and fibre fraction

The results of crude nutrient analyses are summarized in Table 2. The crude protein (CP), crude lipid (CL) and neutral detergent fibre (NDF) content in SJ were significantly lower than in hay. Fibre fraction, acid detergent fibre (ADF), and acid detergent lignin (ADL) levels were significantly higher in SJ than in hay samples.

**Table 2:** Crude nutrients, and fibre fraction in hay and SJ

Parameter	Hay (*N* = 6) (DM: 90.2 ± 1.53)	SJ (*N* = 6) (DM: 90.6 ± 1.59)	*P*-level
CL (%)	1.62 ± 0.414	0.611 ± 0.213	0.002
CP (%)	9.37 ± 1.59	3.50 ± 0.241	0.002
CF (%)	33.7 ± 3.69	38.5 ± 0.964	0.015
NDF (%)	62.8 ± 5.17	57.8 ± 1.89	0.041
ADF (%)	36.3 ± 3.21	45.42 ± 1.74	0.002
ADL (%)	3.8 ± 0.938	5.32 ± 0.311	0.026
NFE (%)	40.0 ± 3.17	43.2 ± 1.78	0.093

Data are expressed as mean ± SD. *CF* crude fibre, *NFE* nitrogen-free extract

## Discussion

To the best of our knowledge, this is the first study investigating the rejection behaviour of horses towards dried SJ in hay. In this study, horses were fed hay ad libitum. Under these conditions, we hypothesized that horses can sort SJ out of hay; however, the findings of this study only partially confirmed our hypothesis. During the observation periods, four out of the six horses did not reject SJ properly, while only two horses were able to refrain from SJ intake during the whole study.

In the literature, one of the frequently mentioned reasons for a reduced rejection of toxic plants in horses is the lack of adequate daily forage provision [14, 15]. Due to hay provision ad libitum, the lack of satiety was ruled out in this study. Terminations due to SJ uptake were equally distributed over the one-hour observation period. Consequently, the uptake of SJ was not correlated with the remaining hay volume (Fig. 2).

Certain plant species produce secondary metabolites, which can act as defence against uptake [16]. In the case of SJ, mainly PAs and sesquiterpene lactones are mentioned as bitter tasting plant metabolites for horses [17]. Petzinger (2011) described a rejection of SJ by horses on pasture due to the bitter taste of fully grown SJ [6]. However, it is assumed that bitter-tasting plant compounds decrease during the drying process, which may reduce the rejection behaviour in horses [6].

In the present study, PA content in dried SJ was comparable to PA levels in fresh SJ material. Our findings are in accordance with the results obtained by Candrian et al. (1984) and Kaltner et al. (2018) who postulated that PA levels remained stable during the drying process [18, 19]. Unfortunately, other bitter-tasting substances were not measured in the present study. For this reason, an evaluation of a possible rejection criterion based on bitter substances was not possible.

In order to investigate the ability of horses to associate a novel food to illness, Houpt et al. (1990) used apomorphine (0.06 mg/kg body weight [BW]) as a rapid-acting emetic to induce food aversions in horses. Apomorphine was injected intramuscularly, directly after novel food consumption or with a delay of 30 min. Findings implicated that horses were able to develop a food aversion when apomorphine-induced nausea occurred immediately after feed intake. No food aversion was observed when apomorphine was injected 30 min after food consumption [20].

Taking the study results of Houpt et al. (1990) into account, the development of food aversion in horses might be linked to acute intoxication with SJ. As SJ intoxication usually occurs as a chronic process with the earliest symptoms manifested weeks or even months after ingestion, the development of food aversion due to nausea seems unlikely in horses.

In addition, horses are probably able to reject their food based on macronutrient contents. Van den Berg et al. (2016) observed that horses discriminate feedstuff in relation to nutrient content, odour, and taste. Horses showed a significantly higher intake of protein-rich (14% vs. 27% CP) but isocaloric feeds, when adapted to the diets for at least four or 5 days. In addition, a higher intake of diets combined with a natural non-caloric sweetener (2.25% of erythritol/stevia mixture) and a higher intake of diets fortified with non-caloric sweet odours (food flavour emulsions: coconut/banana) were noted. In the study by van den Berg et al. (2016) nutrient content was mentioned as the most important criterion for food rejection in horses followed by the food’s taste. Odour had the lowest effect on food intake [21]. In another study, van den Berg et al. (2016) stated that a well-known non-nutritive odour like fresh lucerne reduced the neophobic effect in horses when lucerne odour was added to a novel food [22]. Redgate et al. (2014) tested the preference of horses according to proteins, lipids, and hydrolysable carbohydrates in isocaloric feed. Horses showed a significantly higher preference to a protein-rich diet (CP: 11.6%) and hydrolysable carbohydrate-rich diet (HCO-H: 13.9%) than to fat-enriched rations (CL: 4.3%) after an adaptation phase to each ration fed separately for 3 days [23]. Cairns et al. (2002) investigated the preference for high-energy feedstuff in horses. First, they showed a preference for mint flavour in contrast to garlic flavour in isocaloric rations. Afterwards, a low-energy diet (digestible energy (DE): 9.3 MJ/kg) and a high-energy diet (DE 11.3 MJ/kg) were mixed with each flavour, and the preference was tested. High-energy feedstuff was preferred even when paired with the less palatable garlic flavour after an adaptation period of 10 meals [24].

Furthermore, the findings of the studies previously mentioned demonstrated that horses had a greater preference for familiar diets and that horses showed a strong neophobic response towards unfamiliar diets. A discrimination based on macronutrient content required an adaptation period for at least three meals [21, 23, 24].

In our study, CP and CL levels in SJ were significantly lower than in hay samples. However, we speculated that macronutrient content seemed unlikely to regulate feed intake behaviour as two out of six horses ingested SJ within the first three observation periods.

Besides the rejection behaviour based on taste, odour, or nausea, horses also select their food for structure. As herbivores, horses are highly specialized to consume feedstuffs with high levels of structural substances such as ADF, NDF and ADL [15].

Stainair et al. (2010) monitored the preference of horses for three maturity stages of teff hay. They observed that horses showed a higher preference for earlier headings with a lower NDF and ADF content [25]. Cummins et al. (2014) tested preferences of horses in different maturities and growth under different weather conditions in teff hay and Bermuda grass [26]. Both studies showed that horses did accept hay with higher percentages of ADF, NDF, and ADL such as teff hay. However, they also indicated a strong negative correlation between fibre content (ADF/NDF/ADL) and palatability (ADF: 35.2-41.5%; NDF: 71.1-73.6%; ADL: 3.8-4.4%) [25, 26].

In the present study, fibre fractions such as ADF and ADL were significantly higher in SJ than in the hay samples. However, the ADF content in hay and SJ were found to be lower than that in the study of Staniar et al. (2010) and Cummins et al. (2014) [25, 26]. In contrast, the ADL content in SJ exceeded the ADL content as described by Cummins et al. (2014). An elevated lignin content may have caused the rejection of SJ in two horses but did not explain the intake of SJ in the other four horses.

Another possible explanation for the different SJ sorting behaviour of horses is learned rejection behaviour as a foal. Bolzan et al. (2020) observed that foals learned their rejection behaviour in three steps: 1) explorative phase: the foals tested different plants in small quantities; 2) specialization phase: diversity of plants ingested by the foal decreased and plant intake approached to the feeding behaviour of the mare; 3) stabilization phase: feed intake no longer differed significantly from the dam [27]. In our study, two horses rejected the intake of SJ throughout the whole feeding period. It is possible that these horses had contact with SJ during foalhood.

Limited data is available on the toxic level of SJ in horses. Craig et al. (1991) fed SJ to 12 ponies until lethal intoxication. In the study of Craig et al. (1991) the mean daily intake of PA ranged between 0.79 and 1.7 mg/kg BW. Lethal intoxication occurred between 49 and 406 days after first SJ intake [28]. With respect to the study of Craig et al. (1991) the PA content of SJ used in our study would correspond to an average intake of 454-976 g of the dried SJ material in an adult warmblood horse. Mendel et al. (1988) fed *Senecio vulgaris* cubes, with an average of 233 ± 9.2 mg PA/kg BW, to nine horses over a feeding period of 89-159 days until lethal intoxication [29]. The PA intake by Mendel et al. (1988) corresponded to an estimated daily intake of 1.08 kg dried SJ in our study.

Petzinger (2011) reviewed a tolerable daily intake (TDI) of 1 μg PA/kg BW [6] The TDI by Petzinger (2011) corresponded to 0.57 g of dried SJ plant material intake per day for a horse (600 kg BW).

Furthermore, a no-observed-adverse-effect level was determined for the PA riddelliine in rats, concerning non-neoplastic chronical effects [30]. Using a safety factor of 100, a guidance value derived for non-neoplastic chronic effects of 0.1 μg PA/kg BW per day can be calculated for horses.

Under the premise that horses ingest daily amounts of hay equivalent to 2% of their BW, a 5% contamination for a 600 kg horse may result in an intake of 626.9 mg PA/d. A 10% contamination would lead to an intake of 1252.8 mg PA/d. Both intake amounts exceed the proposed guidance value of 0.1 μg PA/kg BW more than 10,000-fold.

The European Union regulates the handling of contaminated hay as follows. According to regulation (EC) No. 178/2002: ‘[…] feed shall not be placed on the market or fed to any food-producing animal if it is unsafe […]. Feed shall be deemed to be unsafe […] if it is considered to have an adverse effect on human or animal health […]’. Therefore, SJ-contaminated hay should not be placed on the market or fed to food-producing animals. Moreover, regulation (EC) No. 767/2009 complements non-food producing animals: ‘[…] requirements set out in Article 16 of Regulation (EC) No 178/2002 shall apply, mutatis mutandis, to feed for non-food producing animals […]’.

## Conclusions

Two out of six horses were not able to sort SJ out of hay. Two other horses showed an inconsistent rejection behaviour. Only two out of six horses were able to pick out the various amounts of SJ from hay throughout the whole feeding period. The findings of this study showed that the SJ rejection behaviour of horses differs individually. Overall, it is doubtful that horses can completely avoid SJ intake due to rejection, even when hay is offered ad libitum. As even a low level of SJ contamination may lead to chronic intoxication in horses when consumed over a longer period, SJ levels in feed should be generally reduced to the lowest level reasonably achievable (ALARA principle), which is also common practice in the human food sector. Consequently, pastures with SJ plant growth are not appropriate for feed production.

## Methods

### Animals

Six clinically healthy warmblood geldings with a mean (±SD) age of 15 years (±2 years) and an average (±SD) body mass (BW) of 674 kg (±85 kg), owned by the Institute of Animal Nutrition, Nutrition Diseases and Dietetics were housed in individual boxes with straw bedding. Hay was offered ad libitum. Additionally, 50 g of commercial mineral feed was supplemented (Reformin Plus®, Höveler, Münster Germany). Tap water was available at all times. Horses had access to a sand paddock 5 hours daily without any hay provision. All horses were well adapted to the general experimental procedure (general handling by LS and CM, housing and feeding management and video recordings during day and night times).

### Feedstuff

The SJ-contaminated hay was harvested in June 2019 from extensively used meadows in Lower Saxony, Germany. SJ was separated from the hay. Hay without any SJ contamination was harvested in Saxony in June 2019. This hay batch was used for preparation and feeding of hay with defined SJ contamination levels.

### Preliminary work

In order to determine the individual hay intake within 1 hour for the observation period, horses were provided 1 kg of non-contaminated hay. If the hay intake of the individual horse was more than 1 kg per hour, 0.5 kg of hay was added to ensure sufficient hay supply, providing a greater rejection likelihood between hay and SJ (Table 3).

**Table 3:** Amount of hay (kg) and SJ quantity (g) per horse for the one-hour observation period

Horse	Amount of hay	SJ 5%	SJ 10%
1, 2, 5, and 6	1.5	75	150
3 and 4	1	50	100

### Observation period

**Fig. 4:**
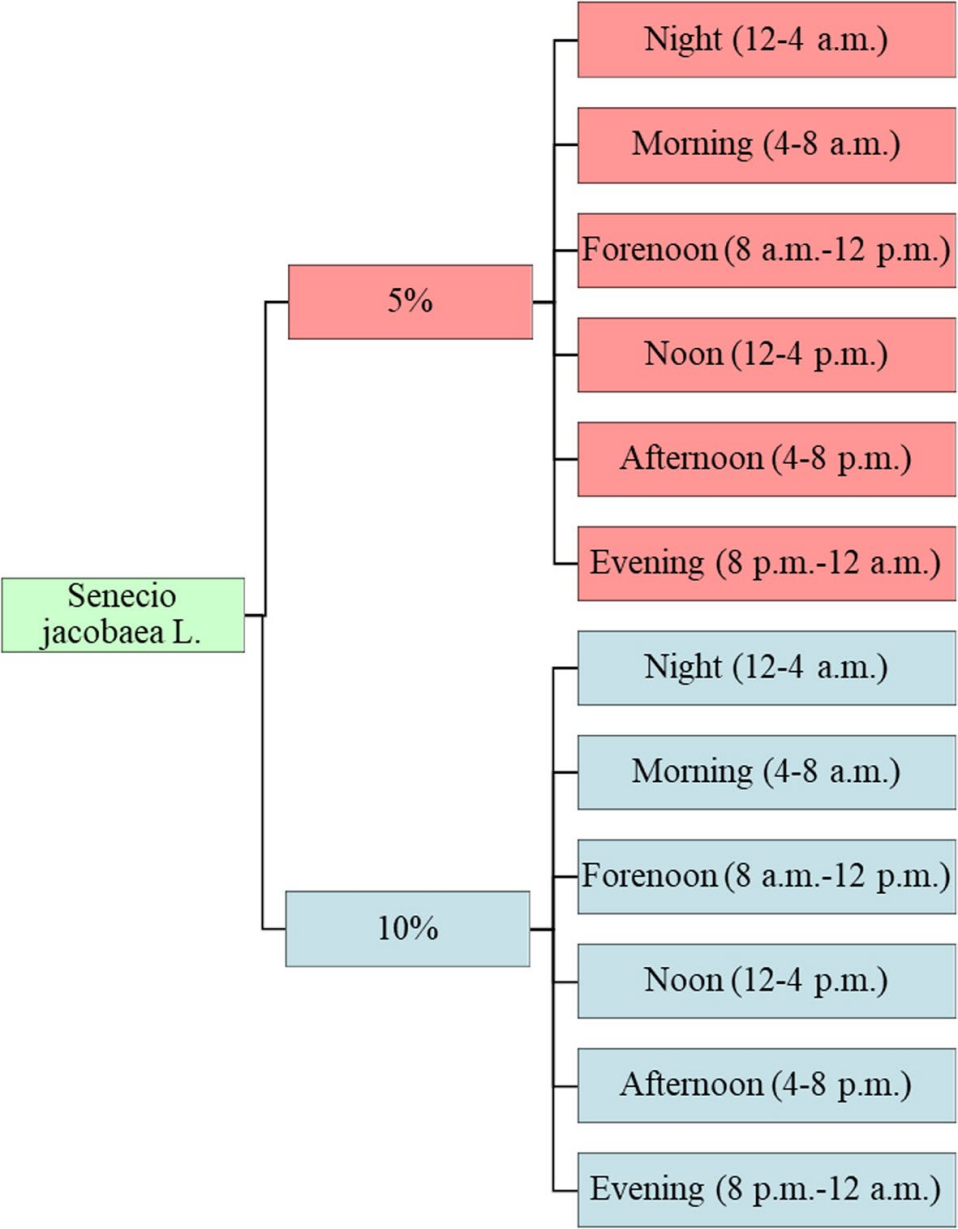
Subdivision of the day into six sections with two contamination levels (5 and 10% Senecio jacobaea L.), resulting in 12 possible observation periods per horse

In this randomized experimental study, the day was divided into six subperiods, wherein each horse was provided a defined amount of SJ-contaminated hay (5% or 10% contamination level). Initially, total study was planned for 48 days, but time period was shortened to 34 days due to the exclusion of horses because of repeated SJ intake (Fig. 4).

SJ was weighed and mixed with the corresponding amount of hay by the same person (Table 3). The SJ-contaminated hay was provided over 1 hour. During the one-hour observation period, horses were observed individually by one or two familiar persons (LS and/or CM). Selected observation periods were additionally recorded by video (Camera: FDR-AX 53, Sony Europe B.V.) by one familiar person (LS or CM) standing next to the feed bucket. Daylight was used for the observation periods during the day. Night time observations were carried out by turning on artificial light. Horses were adapted to this procedure at least 2 weeks before starting with the observation periods.

In the case wherein two stems of SJ were ingested, the experiment was terminated and repeated at another day with at least 2 days of lag in the respective horse. After a second intake of two SJ plants, the horse was excluded from the study.

During the whole feeding experiment, feed residues were removed; hay and SJ were separated, weighed again, and stored for analysis of crude nutrients, fibre fractions and PA-content.

Non-contaminated hay was provided from the same batch during the whole study. Quantity and quality (absence of toxic plants) of non-contaminated hay intake was controlled by weighing and visual controlling.

### Health monitoring

A routine health check, including heart and respiratory rate, rectal temperature, auscultation of the stomach, limb pulsation, colour of mucus membrane and conjunctiva, and alterations of the skin, was performed before and every two to 3 days during the experiment.

### Blood sampling

Blood samples, through single puncture of the external jugular vein, were taken at the beginning and the end of the study. Blood samples were collected in tubes containing either coagulation activator, lithium-heparin or K2EDTA (Monovette, Sarstedt AG, Nuembrecht, Germany) and analysis was performed within 1 hour after sampling.

### Analysis

#### Blood

Serum liver parameters [albumin, total protein, triacylglycerol (TAG), cholesterol, lactate dehydrogenase (LDH), glutamate dehydrogenase (GLDH), aspartate aminotransferase (AST), gamma-glutamyl transferase (GGT), and bile acid (BA)] were measured using an automated chemistry analyser (Roche Cobas C311, Roche Diagnostic GmbH, Mannheim, Germany). Additionally, white blood cells were counted using ADVIA 120 (Siemens Healthineers, Erlangen Germany).

#### Hay

Hay samples (*N* = 6) were taken and analysed for crude nutrients and fibre fractions.

DM was determined after oven-drying (103 °C) to constant mass. Crude nutrients such as CP and CL were assayed by the Weende system (Naumann and Bassler, 2004) [31]. Crude fibre (CF), NDF, ADF, andADL were analysed by ANKOM® (Ankom Technology, Macedon, USA) according to Van Soest et al. (1991) [32]. The nitrogen-free extract (NFE) content was calculated (NFE = DM - (CP + CL + CA + CF)).

#### *Senecio jacobaea* L.

In order to determine crude nutrients and fibre fractions, Weende analysis was performed in randomly collected samples (*N* = 6) of SJ using the methods described previously.

PAs were analysed in the following SJ samples: fresh plant (*n* = 1), dried plant (*n* = 1), SJ sorted out of crib residues of each horse (*n* = 6), and one sample of dried stems (*n* = 1), flowers (*n* = 1), and leaves (*n* = 1) each. All samples were a mix of various plants. PAs were analysed using liquid chromatography with tandem mass spectrometry by the National Reference Laboratory for Mycotoxins and Plant Toxins, Berlin. The total PA content was calculated as a sum of the following retronecine-type PAs: erucifoline, jacobine, jacoline, retrorsine, riddelliine, senecionine, seneciphylline, and jaconine including all their corresponding *N*-oxides as well as their naturally occurring isomers and the otonecine-type PA senkirkine.

#### Sample preparation

For the extraction of PAs, 10.0 g ± 0.1 g of comminuted dried plant material was weighed into a centrifuge tube. A duplicate extraction with a volume of 100 mL aqueous extraction solution containing 0.05 M H_2_SO_4_ was used. For extraction, an ultrasonic bath was used for 15 min, followed by 20 min shaking (overhead shaker). The samples were centrifuged (20 °C, 3800 g, 10 min), the supernatant was passed through a 0.20 μm nylon membrane filter, 20−/50−/100-fold diluted, and subsequently analysed by an external calibration applying a 10-point calibration curve in the range of 0.05-150 ng/mL.

#### Liquid chromatographic analysis

All measurements were conducted on an Agilent 1290 Infinity Series UHPLC system (Agilent Technologies, Santa Clara, USA). Chromatographic reversed-phase separation with 2 μL injection volume was performed on a C18 Hypersil Gold column (150 mm × 2.1 mm; 1.9 μm particle size) with guard column (Thermo Fisher Scientific, Waltham, USA) at a flow rate of 0.3 mL/min and with a column temperature of 40 °C. The binary mobile phase was composed of water as mobile phase A and methanol as mobile phase B, both containing 0.1% formic acid and 5 mmol ammonium formate. The gradient conditions were as follows: 0-0.5 min A: 95%/B: 5%; 7.0 min A: 50%/B: 50%; 7.5 min A: 20%/B: 80%; 7.6 min A: 0%/B: 100%; 10.1-15 min A: 95%/B: 5%.

#### Tandem mass spectrometry

Electrospray ionization tandem mass spectrometry (ESI-MS/MS) data were acquired in the positive ionization mode on a QTRAP 6500 MS/MS system (Sciex, Agilent Technologies, Santa Clara USA). The settings of the ESI source were as follows: source temperature 500 °C, curtain gas 35 psi, ion source gas 1 (sheath gas) 60 psi, ion source gas 2 (drying gas) 60 psi, ion-spray voltage + 5500 V and collision gas (nitrogen) medium.

Two MRM transitions were measured per analyte as follows ([M + H]^+^ underlined, quantifier bold, qualifier plain): Er (350 → 120/2138); ErN (366 → 120/136); Jb (352 → 120/155); JbN (368 → 296/120); Jl (370 → 120/138); JlN (386 → 120/136); Jn (388 → 138/156); JnN (404 → 118/120); Re (352 → 120/138); ReN (368 → 120/118); Rd. (350 → 120/138); RdN (366 → 136/120); St (370 → 120/138); StN (386 → 120/136); Sc (336 → 120/138); ScN (352 → 118/136); Sp (334 → 120/138); SpN (350 → 118/136); Sk (366 → 150/168).

### Data analysis

Descriptive statistical analysis was performed using Microsoft Excel 2016® (Microsoft Corporation, Redmond, USA) and SPSS 27® (IBM, Armonk, USA). All parameters were tested on normal distribution with SPSS 27®. A paired t-test of normally distributed data was performed to compare blood parameters before and after the feeding period. Crude nutrients and fibre fractions were not normally distributed. These data were analysed by a Mann-Whitney U test for unpaired samples to compare hay and SJ samples. Statistical significance was set at *P* < 0.05. Due to the low number of animals and feedstuff, sample size data are expressed as mean ± standard deviation (SD).

## Data Availability

All data generated or analysed during this study are included in this published article.

## Abbreviations

ADF: Acid detergent fibre
ADL: Acid detergent lignin
LDH: Lipid dehydrogenase
AST: Aspartate aminotransferase
BA: Bile acid
BW: Bodyweight
CF: Crude fibre
CLs: Crude lipids
CP: Crude protein
DE: Digestible energy
DM: Dry matter
ESI-MS: Electrospray ionization tandem mass spectrometry
GGT: Gamma-glutamyl transferase
GLDH: Glutamate dehydrogenase
NDF: Neutral detergent fibre
PA: Pyrrolizidine alkaloids
SD: Standard deviation
SJ: *Senecio jacobaea* L.
TAG: Triacylglycerol
