# Correction to: Horses’ rejection behaviour towards the presence of *Senecio jacobaea* L. in hay

**DOI:** 10.1186/s12917-022-03168-w

**Published:** 2022-02-14

**Authors:** Louisa Sroka, Clara Müller, Marie-Lena Hass, Anja These, Sabine Aboling, Ingrid Vervuert

**Affiliations:** 1 Institute of Animal Nutrition, Nutrition Diseases and Dietetics, Faculty of Veterinary Medicine, Leipzig University, Leipzig, Germany; 2 Institute for Animal Nutrition, University of Veterinary Medicine Hannover, Hannover, Germany; 3 Department Safety in the Food Chain, German Federal Institute for Risk Assessment, Berlin, Germany

**Correction to: BMC Vet Res 18, 25 (2022)**

**https://doi.org/10.1186/s12917-021-03124-0**

Following the publication of the original article [1], it was noticed that the figure captions are incorrectly captured. Correct figures and captions are shown below (Figs. 1, 2, 3 & 4).

**Fig. 1:**
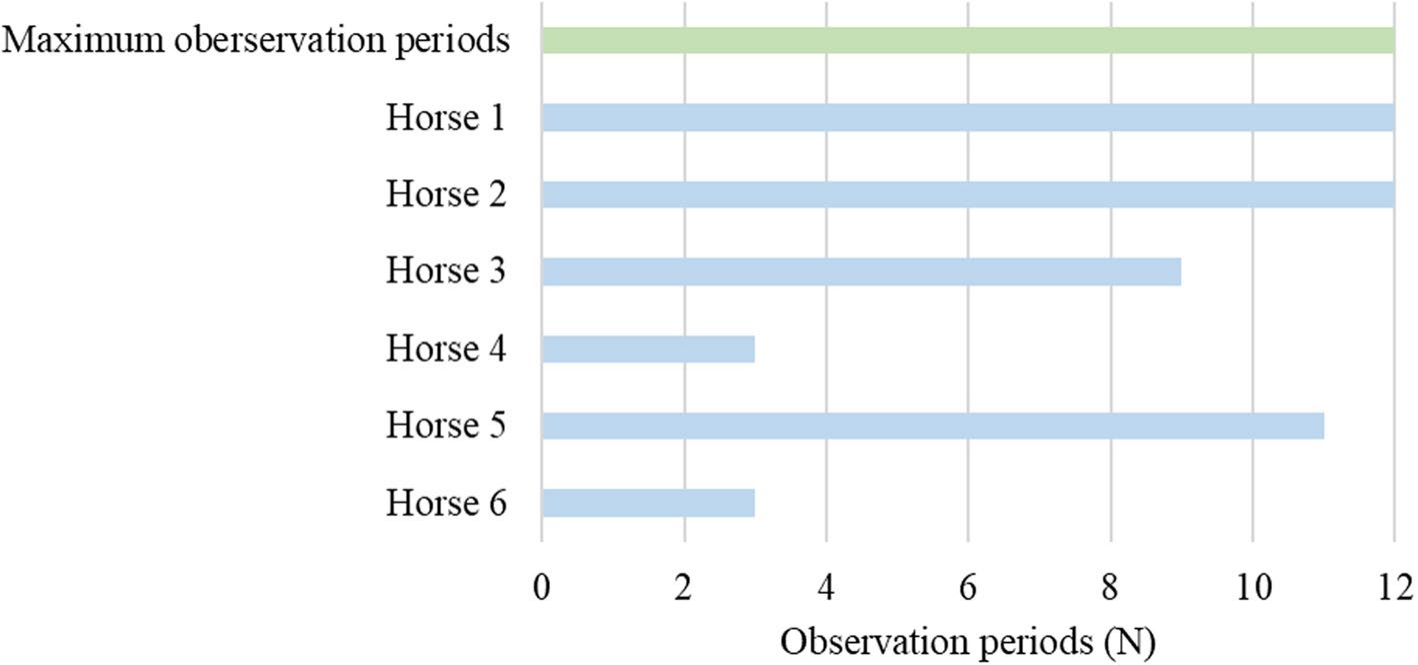
Observation periods of the horses in relation to the maximum possible observation periods (*N* = 12 observation periods per horse). Observation periods below 12 denote an interruption of feeding experiment due to SJ ingestion. Data are expressed individually

**Fig. 2:**
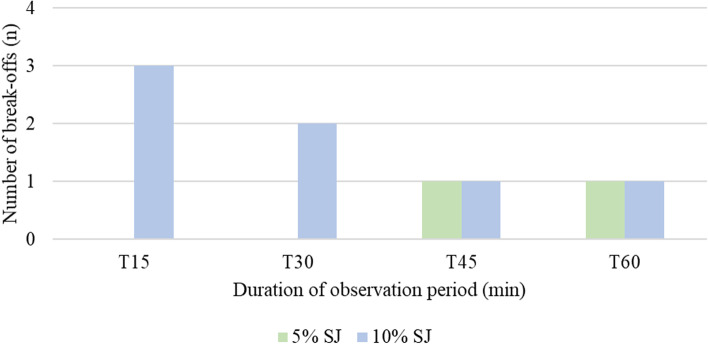
Number of breakoffs in relation to the duration of observation periods (T = time in minutes), breakoffs in total *n* = 9

**Fig. 3:**
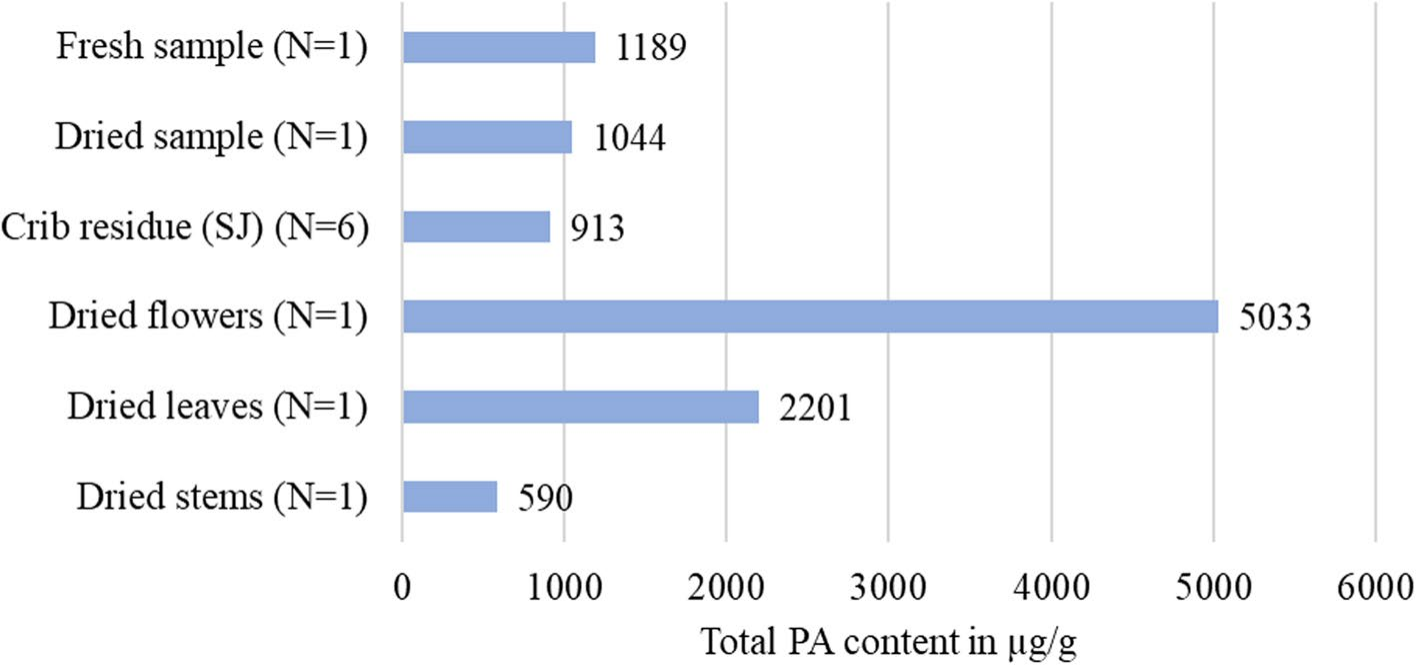
Total PA contents in whole fresh plants or dried plant material. SJ in crib residues and in individual parts such as dried flowers, leaves, and stems. Data are expressed in μg/g (DM)

**Fig. 4:**
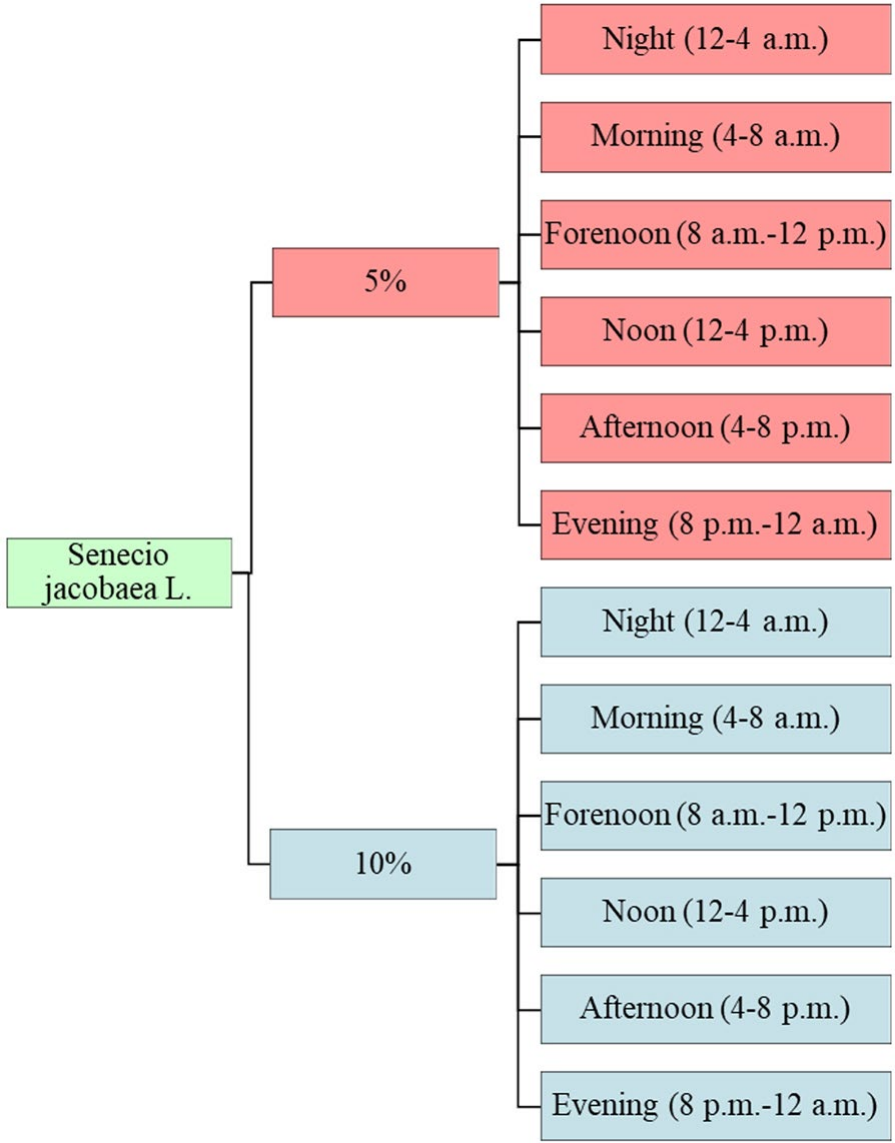
Subdivision of the day into six sections with two contamination levels (5 and 10% *Senecio jacobaea L*.), resulting in 12 possible observation periods per horse

The original article has been corrected.
